# A qualitative examination of policy and structural factors driving care workers’ adverse experiences in long-term residential care facilities for the older adults in Cape Town

**DOI:** 10.1186/s12877-019-1105-3

**Published:** 2019-04-02

**Authors:** Leo Mapira, Gabrielle Kelly, Leon N. Geffen

**Affiliations:** 1 The Samson Institute For Ageing Research, Highlands House, 234 Upper Buitenkant Street, Cape Town, Western Cape 8001 South Africa; 2 Department of Sociology, Faculty of Humanities, 4.35 Leslie Social Science Building, 12 University Avenue, Rondebosch, Cape Town, Western Cape 7701 South Africa; 3 Division of Geriatric Medicine and the Albertina & Walter Sisulu Institute of Ageing in Africa, Faculty of Health Sciences, University of Cape Town, Groote Schuur Hospital, L51 Old Main Building, Observatory, Cape Town, Western Cape 7701 South Africa

**Keywords:** South Africa, Care worker, Long-term care, Older adults, Training curriculum, Functional impairment, Professional mandates

## Abstract

**Background:**

There is lack of adequate training and policy support for employed care workers (CWs) employed in the South African (SA) older persons’ sector. Existing literature neglects the influence of training and policy support on CWs’ experiences in long-term care (LTC) for older adults in residential care facilities (RCFs). We investigated the ways in which CWs’ experiences are rooted in the lack of adequate training and policy support.

**Methods:**

Qualitative data was collected through focus group (FG) interviews with 32 CWs employed in RCFs in the City of Cape Town. Data was also collected using semi-structured interviews with representatives of five RCFs for older adults and four training organisations providing CW training in the City of Cape Town, South Africa.

**Results:**

Despite some positive caregiving experiences, CWs face role ambiguity and experience care work as a ‘career-less job’. They also face poor employment conditions, negative interpersonal relations at work, and role overload. They are not coping with the demands of LTC due to role overload, and lack of basic caregiving skills, coping skills and socio-emotional support. Their motivation to cope and provide quality care is hamstrung by their experiences of role ambiguity, poor employment conditions, negative interpersonal relations at work, and lack of career growth opportunities.

**Conclusions:**

Findings suggest that CWs’ experiences derive from the policy and structural context of caregiving. Policy inadequacies and lack of structural support create conditions for adverse conditions which negatively impact on CWs motivation and ability to cope with the demands of LTC. Lack of policy implementation presents structural barriers to quality LTC in the older persons’ sector. Implementation of policies and systems for professionalising care work is long overdue.

## Background

LTC systems are required in Sub-Sharan Africa due to the growing population of functionally dependent older adults [1]. One of the key elements of an older-person-centred LTC system is a competent care workforce with specialised training and support, including nurses and CWs [2]. The distinction between health and social care is not easy to define as the two overlap and both can be seen to contribute to health and social gain [3]. The ambiguous roles of the LTC workforce has been subject of much research and debate [4–7]. For example, nurses experience their work as highly complex and unpredictable [4, 6, 7]. They have difficulty defining and limiting their roles because they have all-embracing roles [5, 6]. CWs also face the challenge of developing and consolidating their professional role in LTC [8, 9]. They define their role in terms of what they are *not* allowed to do, particularly nursing work [7]. South African literature indicates that CWs in private homes may be task-shifting upwards, doing more nursing-type care [10]. They are required to provide holistic care, including performing nursing tasks such as providing antiretroviral and tuberculosis treatment [11]. They have no clear scope of practice, and parameters and legal boundaries [9]. There is need to examine of the roles of the LTC workforce to ensure greater coherence and clearer roles, responsibilities and accountability [4, 5].

An older person-centred system of LTC require skilled CWs, but CWs lack skills training and support [12]. Public policy should address the need for standardised training programmes for CWs, so that they are equipped with multiple skills [9]. The African Union promotes public policies on training and support so that CWs have skills and knowledge commensurate with their roles [13]. Considering this South Africa enacted the Older Persons Act of 2006 and its Regulations and set policies through the Health and Safety Sector Education and Training Authority (HWSETA) to provide for the training and support of CWs. Career pathways opportunities and incremental accreditation/credentialing for CWS need to be implemented [14]. Resource constraints and administrative and management problems continue to be barriers to implementation [14]. Despite these policies care work remains precarious, characterised by ill-defined professional mandates of CWs and, inadequate and inconsistent training [9], and lack of social support for CWs [15].

CWs’ experiences occur at three levels, namely: the *caregiving context* (micro)*, organisational context* (*meso*)*, broader societal context (exo)* and *policy and structural context (macro)* [16, 17]. Within the *caregiving context*, caregiving is inherently burdensome and stressful due to the suffering and dying of care recipients [18], the complexity of dementia care [19], and the emotional demands of caregiving [20]. Social support should be provided for maintaining the psychological wellbeing of both care recipients and their formal carers [21]. At the *meso* or organisational level, workplace-based social support, interpersonal relations, compensation and working conditions impinge on CW stress, and lack of job satisfaction [22–25]. In turn, job satisfaction influences CWs’ caring motivations and the quality of care they provide to older adults [26, 27]. The *broader societal context* of caregiving involves structural issues such as gender, ageism, and external support systems that influence CWs’ interaction with care recipients [16]. For example, stigmatisation of CWs in wider society could demotivate CWs [28]. At *macro-level* policy and structural factors impinge on CWs’ experiences [16]. For example, the lack of professional recognition of CWs by the state can create difficult conditions for CWs to perform their duties [29].

South African literature on caregiving is largely framed within the context of informal caregiving, including caregiver burden, training and social support for informal caregivers [30, 31]. The scant but growing literature on formal caregivers (CWs) focuses on the *organisational context of caregiving*, including themes such as work-related stress and social support [32, 33]. However, comprehensive studies on the policy and structural context of elder care are scarce [9, 10, 29]. In this context, little is known about the impact of the current policy and structural context of caregiving on CWs’ experiences in residential care facilities for older adults. National policies can improve the provision of professional training, regulation of CWs’ roles, and the provision of psychosocial support to enhance CWs experience and quality of care [8, 34, 36]. We contend that CWs’ experiences in RCFs, including their ability to cope with the demands of LTC, are more entrenched in the policy and structural context of LTC than the caregiving, organisational and societal contexts. We examined the ways in which CWs’ experiences are deeply rooted in the policy and structural context of caregiving - in particular, CW training and development structures, institutional support for care work, and long-term care funding*.*

## Methods

Existing literature shows that policy and structural factors influence CWs experiences. We then took an inductive qualitative approach to explore the ways in which the policy and structural context of LTC influence CWs’ caregiving experiences, and ability to provide quality elder care. In-depth qualitative interviews were conducted with representatives from five RCFs, three nursing agencies and four training organisations. Focus Group Interviews (FGIs) were held with CWs recruited through RCFs. Interview Guides replete with open-ended questions were used in both Individual Interviews with representatives of organisations and FGIs with CWs. Follow-up questions were improvised during the interviews to elicit more in-depth information where possible. There are no interviewer characteristics that could influence participants responses during the interviews. The FGI interviews were electronically recorded with the consent of participants. The average duration of FGI Interviews was one hour.

We used a purposive sampling technique in which participants were selected using the principle of maximum variation to include participants with a variety of training experiences and work experiences. We first approached managers of RCFs for permission to conduct research with their CWs. The managers helped with advertising the study among all CWs employed at their RCFs. They also assisted with setting up meetings with CWs, during which the Researcher explained the objectives of the study. CWs who were willing to participate recorded their names, training and qualifications. We then selected forty participants from the list. A total of forty CWs were invited to participate, but eight chose not to participate.

Thirty-two CWs at four RCFs in the city of Cape Town Metropole were interviewed between June and July 2017 at their workplaces. No other person was present in the interview room for confidentiality purposes. Participants were all female, aged between twenty-three and fifty years. They all came from low income neighbourhoods in the City of Cape Town. With the exception of one participant, all possessed a formal post-secondary Home-Based Care qualification. Participants’ years of service in formal caregiving ranged from three to nine years.

The study received ethical approval from the Human Research Ethics Committee of the University of Cape Town’s Faculty of Health Sciences (*HREC REF: 160/2017)*. CWs voluntarily participated in the study and were guaranteed the right to withdraw from the study. The results of the study were shared with RCF representatives, but the actual information obtained in the interviews with (CWs) was not shared with RCF representatives for confidentiality purposes. The results of the study were also presented to CWs who participated, and their comments were addressed.

The data was analysed using the *Miles and Huberman* approach to thematic analysis [35]. We first formulated an a priori thematic coding framework based on existing theory and existing evidence. The coding framework guided the data analysis process, but other themes emerged inductively from data. The coding strategy included first-level coding for reduction of data into meaningful themes (first-level coding). This was followed by the identification of coherent clusters and hierarchies of *data* within the first-level codes. Finally, an iterative process of identifying relationships, patterns and explanations was implemented. Analysis of data iteratively conducted by the main researcher was done using Nvivo 11 software.

## Results

### Role ambiguity and career growth

CWs’ role expectations do not align with their actual scope of work. They are generally regarded as non-medical social care personnel in the South African primary care system, but they understood their role in primary care as more or less part of the nursing profession. As a result, they experienced role ambiguity. This is exhibited by their reference to themselves as ‘nurses’ in interviews. Their lack of a clear professional identity is perhaps the most recurrent theme in this study. The following response from a participant explains CWs’ role ambiguity:

*I am a nurse. It’s not like a caregiver (CW) for me. I am a nurse because in private hospital I got a lot of experience its only here where you work as a caregiver because in private hospital you work with the ENA and staff nurses and you get a lot of experience. Not only observations, I did injections, and all the basics. I was doing all that, but here you are not allowed to do that…but in private agencies…* (CW 5, FGI 2, LTC Facility 1, July 2017).

Additionally, CWs rarely have opportunities for enhanced roles beyond social care. RCFs that do provide limited opportunities for enhanced roles – including undertaking basic medical tasks – are largely low-income facilities that lack sufficient professional nurses to provide medical care. In general, CWs feel that RCFs are limiting their role to the most rudimentary social care tasks such as assistance with activities of daily living. This is creating despondency among CWs, as shown by their contempt for professional nurses and care managers, who they saw as limiting them to the most basic care tasks.

In terms of public perception of care work and CWs, CWs role is largely perceived to be different from nursing. CWs are stigmatised as *‘bum cleaners’* (CWs, FGI 1, LTC Facility 1, June 2017). This derogatory term is instructive of the lack of appreciation of CWs’ role in elder care by some members of the public. The stigma attached to care work derives from the public’s understanding that care work is an unpleasant, demeaning and poorly-paid generalist job that requires little skill or experience. However, data also indicate that CWs are misconstrued as nurses in some local communities. They are called upon to provide informal basic ‘nursing services’ to community members.

Most participants partook in care work as a ‘springboard’ into the nursing profession. They reported that they do not have opportunity to progress in the health and social services sectors, and that they feel ‘stuck’ in care work, as one CW describes:

*I want to go further not just being a caregiver but I’m here. I’m stuck, I can’t go study further* (CW 4, LTC Facility 3, July 2017).

Care work is currently experienced by CWs as a ‘career-less job’. They cited many barriers to career development in the health and social services sectors, unaccredited training, unrecognised CW training certificates and financial challenges.

CWs’ experiences of role ambiguity, lack of a clear professional identity and lack of opportunities for career progression seem to be sources of dissatisfaction among most CWs. They are dissatisfied with care work, and it appears that many remain in care work largely because of the lack of opportunities in other fields.

### Employment conditions of CWs

CWs do not enjoy standard employment conditions. Outsourcing of care work to nursing agencies by two RCFs led to loss of relatively stable and permanent employment among CWs who were initially employed directly by the two RCFs. Outsourcing has also led to reduction of some CWs’ wages and benefits. All CWs deplored their temporary employment status, poor remuneration and lack of employment benefits, although those employed directly by RCFs had relatively stable employment and better working conditions. The average monthly wage for CWs is R4 000 per month (US$ 318, 40), with those employed through nursing agencies earning as little as R3 000 per month (US$ 238,80). Many CWs earn below the national minimum wage of R3 600 per month (US$286, 82). The current minimum wage itself is inadequate for workers’ cost of living, and is currently being reviewed by the South African Department of Labour [28] .

CWs complained about arbitrary deductions on their salaries, and non-payment of overtime work by nursing agencies. Those employed directly by RCFs also reported arbitrary wage deductions by the RCF management, for example when things go missing at RCFs. The perceived lack of transparency in the administration of remuneration is demotivating CWs. One CW said:

…*.because I put two days extra and I don’t get extra money. It’s not fair. Next month I won’t say yes because I did not receive last month’s two days money for overtime. Even if it’s in my heart, they cannot take advantage of me being a carer. So, you care more but you don’t get more*. (CW 3, LTC Facility 5, July 2017).

CWs employed through nursing agencies were bewildered by disparities in remuneration, benefits and employment status between them and their counterparts despite doing the same work. Poor employment conditions and perceived lack of administrative transparency by nursing agencies and RCFs is creating a sense of dissatisfaction among CWs.

### Work-related stress and coping

LTC is inherently stressful for CWs but there are additional sources of subjective stress and burnout reported by CWs, including work/role overload. Although participants worked 180 h per month, as regulated by the Basic Conditions of Employment Act, subjective experiences of stress and burnout are partly due to having too many care recipients.

At one low-income RCF CWs reported that the caregiver-patient ratio was 1:10 during the day and 1:20 during the night:

*….during the day its fine, but not during the night. So, at night its twenty residents for one CW…some residents (care recipients) are always going up and down. They never sleep …. you can’t even go to tea time at night*. (CW 3, LTC Facility 5, July 2017).

Subjective work-related stress among CWs was also due to their lack of coping skills. Most CWs employ coping styles that may be dysfunctional, including *avoidance-coping*, *emotion-focused coping* and the reliance on *personal attributes of care* such as patience and tolerance. Some simply ‘put on a smile’ and go back to work; an avoidance-type of coping. Others react to stress with indifference, which is also an avoidance-type coping strategy. Such maladaptive emotion-focused coping is exemplified by one CWs’ response:

….*You cry, wash your face and smile…no one talks to you and asks how you feel about it, and say how can we solve this to please you…. because in five minutes time you must go back…* (CW 4, LTC Facility 3, July 2017).

Only a small minority of CWs employed positive coping strategies such as the strategy of *switching between care environments or care recipients* to avoid dealing with patients or certain care tasks that are stressful. For example, one CW said:

*I learnt from M is that she works for a time period in one ward and then suddenly there M goes to another ward…she knows that this stuff takes a strain on your body…. and that’s how she keeps on going for all these years* (CW 5, FGI 2, LTC Facility 1, June 2017).

Difficulties in coping also emanate from skills gaps in areas CWs are supposedly trained, including dementia care skills, dealing with falls and fractures, and first aid. Some RCFs even employ CWs with no appropriate formal CW training, raising doubts about their caregiving skills. At one RCF, most CWs did not have the formal dementia care training required for dementia care environments. For example, a CW at one RCF said:

*…not all the CWs….have the knowledge of how to work with dementia people, and even I don’t have it, but what I do use is my common sense….*. (CW 4, LTC Facility 5, July 2017).

Skills upgrades and socio-emotional support that provide additional coping skills are mostly unavailable to most CWs, making caregiving more stressful and challenging for CWs. In some RCFs, socio-emotional support is rare, and it is only provided at RCFs in extreme cases of bereavement and severe abuse of CWs by care recipients.

### Subjective experiences of being unappreciated

CWs reported that they are not being acknowledged and unappreciated in RCFs. They reported that management and other professionals ‘look down’ on CWs, and that they are not supportive. They allegedly do not appreciate the essential role that CWs play in caring for older persons. For example, one CW had this to say:

*It’s like what happened yesterday. The doctor phoned and there wasn’t any staff nurse around…..I could have told the doctor what he wanted to know in the first place. So to me why did the doctor not tell me why he phoned?* (CW 4, LTC Facility 5, July 2017).

Additionally, CWs reported that they are overlooked by care recipients’ families when they visit RCFs, and are sometimes accused of stealing from care recipients:

*For me it’s sometimes the families. They don’t greet us. They put us so low because they think it’s easier to care but they don’t know what a carer is, and what we are going through. They go to sisters (nurses) because to them we are nothing*. (CW 5, LTC Facility 5, July 2017).

Real and perceived lack of formal and informal acknowledgement of CWs’ effort and contribution appear to negatively affect CWs’ confidence and motivation to execute their duties.

## Discussion

Our results indicate that CWs struggle to cope with the demands of LTC due to many factors, including overwork, lack of basic caregiving and coping skills and inadequate socio-emotional support. Their motivation and ability to cope with and provide quality care in LTC settings is also hamstrung by poor employment conditions, real or perceived negative interpersonal relations at work, role ambiguity and lack of career growth opportunities.

LTC for older adults is intrinsically stressful, but dynamics inherent in the caregiving context do not entirely explain CWs’ experiences. We argue that CWs’ experiences are deeply rooted in the broader policy, institutional and structural context of LTC for older adults.

Role ambiguity derives from the lack of clearly defined professional mandates. CWs’ mandates and conditions of service remain unregulated. The Policy on Social Service Practitioners proposed in 2013 to designate and regulate care work as a social service practice, to develop an occupational framework for CWs, and establish a regulatory body for care work, is yet to be implemented [36, 37]. Such policy inadequacies put CWs in the position of working without clear professional mandates and conditions of service, and professional prestige. In the absence of clearly defined professional mandates, current CW training exacerbates role ambiguity and CW despondency. CW training programmes are designed to train CWs on basic medical/nursing procedures and some CWs undertake a *nurses’ pledge* that is meant for professional nurses.

Our findings also show that the role of CWs in LTC is largely not ambiguous in the eyes of the public, although there are some indications that CWs are misconstrued as nurses by some members of the public. Stigma, lack of acknowledgement of CWs role, and limited support both within the older persons’ sector and within South African society appears to be a source of CWs’ despondency. This confirms that challenges experienced by CWs are partly influenced by contextual factors within South Africa [15], which in turn derives from lack recognition and policy support by the government [29]. This situation is an afront to the well-being of care recipients. Thus, these findings confirm that the delineation of specific job functions of CWs is one of the greatest challenges CWs face in developing and consolidating their professional role [8] and in providing quality long-term elder care [9].

CWs’ lack of opportunities for career growth is also the result of policy inadequacies in LTC. Care work is currently not a professional occupation as it falls outside an occupational framework and structured career pathways for CWs do not exist [38]. Secondly, professional training is inadequate, inconsistent and in many cases unaccredited [9]. CWs’ skills certificates are largely not recognised for career progression within the health and social services sectors. This has resulted in care work as a ‘career-less’ job which is another source of their dissatisfaction.

This study provides anecdotal evidence of task-shifting observed in HCBC care environment by prior research, particularly CWs assuming nursing tasks [10]. The practice is an attempt to cope with dwindling long-term care funding. Task-shifting can positively contribute towards job satisfaction among CWs by making their jobs more ‘meaningful’. However, there are serious legal implications as the Older Persons’ Act and its Regulations require that nursing services in RCFs be delivered by professional and registered nurses. Against the backdrop of poor, unaccredited and inconsistent CW training, and the lack of social support for employed CWs the quality of nursing care provided to older adults by CWs is highly suspect. Under the current conditions the task-shifting puts the health and well-being of older adults at risk.

This study confirms that CWs face difficult employment arrangements such as irregular employment, and poor remuneration [10, 38]. Care work is generally a poorly paid work, but poor employment conditions are influenced by the structure of the LTC system. Seventy-four per cent of the 605 LTC facilities are government-subsidised non-governmental organisations and due to insufficient public expenditure on LTC [39] are underfunded. RCFs face significant financial constraints as the subsidy has not been sufficient to cover the costs of providing care [40]. This, for example, explains the high caregiver-patient ratio that exposes CWs to work-related stress and burnout. Driven by insufficient funding outsourcing creates demotivated and insecure CWs by giving rise to casual, informal, non-permanent and are poorly paid CWs.

CWs’ inability to cope with the demands of elder care is also influenced by the current training framework. CWs are supposed to be trained in geriatric care, interpersonal relationships and cultural diversity [38], but in practice they receive minimal, inconsistent, and unaccredited training [9]. Poor skills training emanates from the lack of a comprehensive and standardised national training curriculum, which creates conditions for the poor training provided by training organisations [9]. Training is positively associated with coping [41], but poor training is making CWs’ work more stressful. Ongoing socio-emotional support can provide CWs with additional coping skills [42], but these are rarely available or accessible to CWs. These findings also confirm that CWs generally do not receive reciprocal care and support from employers (RCFs), the government and the broader South African society [15].

Care work is currently precarious, negatively affecting the quality of life of both CWs and their care recipients. Findings suggest the need for policy support and workplace-based support for CWs to directly address the needs of CWs and their care recipients. Policies need to be put in place to promote a competent, motivated and committed workforce that can cope with the demands of LTC. Professional empowerment of CWs can be achieved by clarifying, formalising and regulating their professional mandates. There is need for a career development framework, including specialised and standardised professional training curriculum, structured career pathways, and the provision of opportunities and resources for regular professional support. A registration and licensing system is also important for monitoring the standard of care provided by trained CWs. These measures could give CWs a sense of prestige and career identity.

## Conclusion

Results overwhelmingly show that CWs experience role ambiguity, experience care work as ‘careerless job’, experience negative workplace relations, and face poor employment conditions which negatively impact on their motivation and ability to cope with inherently stressful LTC. This study highlights the role that the policy and structural contexts of caregiving play in explaining CWs’ experiences in LTC. CWs’ experiences derive from the lack of policy and structural support, confirming that the lack of recognition of CWs by the government creates difficult conditions for CWs to perform their duties [29]. This study also confirms that training and unregulated professional mandates remain structural barriers to the provision of quality care by CWs [9]. While it is important for RCFs to address some of the challenges facing CWs, broader policy issues need to be tackled by the government in consultation with all stakeholders. The South African Department of Social Development need to speedily implement a Caregiver Register provided for by the Older Persons’ Act, a professional body for CWs proposed by the Policy on Social Service Practitioners 2013, and to implement innovative LTC funding mechanisms.

## Abbreviations

CW: Care worker
FGIs: Focus group interviews
LTC: Long-term care
RCFs: Residential care facilities
